# Elevation of sputum matrix metalloproteinase-9 persists up to 6 months after smoking cessation: a research study

**DOI:** 10.1186/1471-2466-10-13

**Published:** 2010-03-14

**Authors:** Noora Louhelainen, Harri Stark, Witold Mazur, Paula Rytilä, Ratko Djukanovic, Vuokko L Kinnula

**Affiliations:** 1 Department of Medicine, Division of Pulmonary Medicine, University of Helsinki, Helsinki, Finland; 2 Finnish Institute of Occupational Health, Helsinki, Finland; 3 University of Helsinki, Helsinki, Finland; 4 Department of Medicine, Division of Infection, Inflammation and Repair, Southampton General Hospital, Southampton, UK

## Abstract

**Background:**

Smoking cessation is the best possible way to prevent the progression of smoking related airway diseases. However, the effect and time scale of smoking cessation on airway inflammation/remodelling are largely unknown. This prospective study evaluated the effects of smoking cessation on induced sputum (IS) neutrophils, matrix metalloproteinases (MMP-7, -8, -9) and tissue inhibitor of metalloproteinase-1 (TIMP-1).

**Methods:**

A total of 61 subjects participated in the study; 17 stopped smoking for 3 months and 9 for 6 months. The proportion of IS neutrophils and the levels of MMPs and TIMP-1 by ELISA were determined at baseline and at 3 and 6 months after cessation.

**Results:**

In the smokers, baseline IS neutrophils, MMPs and TIMP-1 were significantly higher compared to non-smokers. Levels of MMP-7, -8 and TIMP-1 decreased nearly to those of non-smokers but the levels of MMP-9 increased significantly from the baseline of the same subjects at 3 months after cessation (p = 0.009) with no significant decline at 6 months after cessation.

**Conclusions:**

Sputum MMP-9 remained elevated after 6 months of smoking cessation, which may contribute to ongoing lung damage typical of COPD.

## Background

Chronic obstructive pulmonary disease (COPD) is one of the most important causes of death all over the world [1]. Smoking is the single most important risk factor of COPD and smoking cessation the only intervention that is able to reduce the disease progression [2,3]. Smoking asthmatics have been excluded from most investigations, but it is known that the inflammatory profile in these subjects is very similar to COPD, with one of the most important features being the influx of neutrophils to the airways in asthma [4]. There is a remarkable overlap in many features in smokers with asthma and subjects with COPD; recent studies even suggest that the majority of older people may suffer from both asthma and COPD [5]. It is not known how the smoking cessation exactly changes the airway inflammatory processes in chronic smokers with or without asthma or COPD.

The very few available studies appear to indicate that airway inflammation and oxidative stress persists after smoking cessation over months in subjects with chronic bronchitis and COPD [6-8]. Evidence of ongoing inflammation has been documented via the increased levels of neutrophils and eosinophils in bronchoalveolar lavage (BAL) fluid, CD4+ and CD8+ cells in bronchial biopsy specimens and eosinophilic cationic protein (ECP) in sputum. The majority of the studies assessing the effects of smoking cessation on airway inflammation have been cross-sectional and there is a lack of prospective investigations.

Extracellular matrix (ECM) deposition is one pathological form of the tissue remodelling detected in the airways [2,9,10]. Matrix metalloproteinases (MMPs) have an important role in the breakdown of ECM and they are considered as biomarkers of tissue damage in several smoking related lung diseases [10-12]. Over 30 MMPs have been characterized, many of which are activated by smoking and/or oxidative stress [13,14]. The levels of MMP-8 and -9 have been shown to be elevated in both experimental emphysema and COPD [10,15,16]. Levels of MMPs on sputum, mainly MMP-9, are also elevated in asthma when compared to the controls [17]. TIMP-1 (tissue inhibitor of metalloproteinases-1) is the major endogenous inhibitor of MMP-8 and -9, and the levels of this protein are elevated in COPD [15]. MMP-7 is produced by alveolar macrophages, breaks down elastin and has an important role in the maintenance of innate immunity proteolytically activating anti-bacterial peptides in the lung [12]. Its role in asthma and COPD remains to be clarified [10,17].

Induced sputum (IS) collection is a non-invasive method for assessment of airway inflammation in the airways. In the future, useful biomarkers may also be identified in IS, to permit accurate determination of the many phenotypes of smoking-related diseases [18,19].

This prospective study was undertaken to assess if smoking cessation is associated with changes in numbers of neutrophils, and the levels of MMP-7, -8, -9 and TIMP-1 in induced sputum specimens. Inflammatory markers were determined at baseline and at three and six months after quitting of smoking to better understand the time course of smoking related alterations and possible temporal differences in the levels of the MMPs. The results were also compared to the corresponding values obtained from non-smoking controls. This is the first prospective study where the effects of smoking cessation on human MMPs have been assessed.

## Methods

### Subjects

Originally, 61 volunteer individuals consisting of 25 asymptomatic smokers, 15 smokers with mild asthma defined according to GINA [20], 7 with chronic bronchitis (previous Stage 0 COPD by GOLD criteria i.e. symptomatic smokers) and 14 subjects with GOLD Stage 1-2 COPD [1,21] were recruited to the study. There were 11 asthmatics and two subjects with COPD using inhaled steroids (daily doses ranging from 400 to 2000 μg). All the asthmatics and five subjects with COPD had short-acting β_2_-agonists as rescue medicine. The subjects were advised to terminate smoking by a research nurse and were provided available pharmaceutical therapies i.e. nicotine products and bupropion to support their abstinence, based on the instructions by the physicians. Smoking cessation was confirmed by exhaled carbon monoxide analysis. The group of non-smokers included never smokers or ex-smokers who had stopped smoking at least 20 years previously. Three of the smokers, three of the COPD patients and 6 asthmatics revealed at least one positive reaction in the skin prick test analyses for common aeroallergens (birch, grass, mugwort, *Cladosporium herbarum*, cat, dog, horse, and the house dust mite *Dermatophagoides pteronyssinus*). The subject characteristics of the smokers and non-smokers, who were also included in the study, are given in Table 1.

**Table 1:** Subject characteristics of all the patients recruited for the smoking cessation.

	Non-smoking controls	Asymptomatic Smokers	Smokers with smokers	Chronic asthma bronchitis/COPD
**Subjects (n)**	30	25	15	21
**Female/male**	7/23	15/10	9/6	14/7
**Age yrs**	56 ± 8	42 ± 12	42 ± 12	56 ± 10
**Pack-yrs**	4 ± 8	23 ± 15	22 ± 14	39 ± 15
				
**Post-bronchodilator**				
FVC L**	4.9 ± 0.71	3.9 ± 0.60	4.2 ± 0.70	3.0 ± 0.66
FVC % pred	102 ± 9	99 ± 14	103 ± 17	102 ± 38
FEV1 L**	4.0 ± 0.65	3.2 ± 0.49	3.5 ± 0.55	2.1 ± 0.72
FEV1% pred*	104 ± 12	97 ± 8	99 ± 14	79 ± 8
FEV1/FVC	82 ± 4.4	83 ± 1	80 ± 9	69 ± 15

Data presented as mean ± SD or median (range). FVC: forced vital capacity; % pred: % predicted; FEV1: forced expiratory volume in one second; *: p < 0.01; **: p < 0.001. #: baseline levels at once after smoking cessation.

The study protocol was accepted by the Ethics Committees of the Helsinki University Hospital and the Southampton University Hospital, and it was performed according to principles of Helsinki Declaration [22]. The subjects provided a written permission for participating in the study.

### Lung function tests

Standard spirometric values (Medikro M 904, Kuopio, Finland) were performed according to ATS/ERS recommendations [23]. Reference values compiled by Viljanen et al. [24] for Finnish population were used.

### Sputum induction

As recommended by the ERS Task Force, the subjects inhaled hypertonic saline in order to provoke IS production [18]. Four volumes of dithioerythritol (DTE, Sigma, Germany) were added as the weight of the sample. The supernatant was frozen at -80°C for biochemical analyses. Cell viability was studied by Trypan blue in a Burker chamber.

Cytocentrifuge preparations were made by Cytospin (Shandon Cytospin 3) and centrifuged at 450 rpm for 6 minutes. The slides were stained by May-Grunwald-Giemsa-staining (Merck, Germany) for cell differential counts with 400 cells being counted from each slide. If the samples had less than 70% of squamous epithelial cells they were accepted for further assessments. The slides were frozen at -20°C.

The IS levels of MMP-7, -8, -9 and TIMP-1 were studied at the onset of smoking cessation and three and six months later. The numbers of neutrophils were assessed at the same time points.

### MMP-7, MMP-8, MMP-9 and TIMP-1 analyses

MMP-7, MMP-8, MMP-9 and TIMP-1 concentrations were analysed by commercial ELISA kits (Amersham Biosciences, Cardiff, UK) according to the manufacturer's instructions. The detection limits were 0.016 ng/ml for MMP-7, 0.032 ng/ml for MMP-8, 0.6 ng/ml for MMP-9 and 3.13 ng/ml for TIMP-1.

### Statistical methods

The data are given as means (standard deviation) or medians (range). Values between groups were analysed with Mann-Whitney U and Kruskal-Wallis tests. The differences in MMP levels between two time points (baseline vs. three months after smoking cessation, baseline vs. six months after cessation, three months vs. six months after cessation) were analysed by Wilcoxon signed rank test. The data were analysed by SPSS for Windows 15.0 (SPSS Inc., Chicago, IL, USA) and a p-value < 0.05 was considered statistically significant.

## Results

There were 17 subjects who successfully stopped smoking for a time of three months and 9 for half a year. Characteristics of these subjects are given in Table 2. Of these 17 individuals, six were asymptomatic smokers, four had mild asthma and seven were suffering from chronic bronchitis or COPD (one with previous GOLD Stage 0 and six with Stage 1 i.e. mild COPD). Four asymptomatic smokers, four asthmatic individuals and one subject with COPD succeeded in quitting smoking for six months. All subjects who completed the study had measurements at baseline and at 3 and 6 months. Due to the high number of dropouts, the three subgroups became small. The subjects in these subgroups were either asymptomatic or symptomatic smokers, or had mild asthma or mild COPD. Therefore, in the MMP analyses these three groups were assessed as a single group. The inflammatory profile in the sputum of these smokers revealed neutrophil predominance. In smokers and in non-smokers the percentages of neutrophils and the total count for neutrophils were 54.5 ± 18.7% vs 30.0 ± 12.0% and 2.2 ± 3.1 (10^6 cells/ml) vs 0.42 ± 0.55 (×10^6 cells/ml), respectively. The levels of the MMP-7, -8, -9 and TIMP-1 in smokers and non-smokers were 12.7 ± 11.5 ng/ml vs 1.7 ± 0.96 ng/ml, 91.7 ± 113.5 ng/ml vs 22.0 ± 29.3 ng/ml, 50.1 ± 63.2 ng/ml vs 21.9 ± 11.9 ng/ml and 1383.3 ± 1322.2 ng/ml vs 822.3 ± 582.7 ng/ml, respectively. Baseline proportion of neutrophils and the levels of the MMPs and TIMP-1 were significantly higher in smokers compared to those of non-smokers in a cross sectional setting (p = 0.021, p = 0.014, p = 0.001, p = 0.02, p = 0.006, respectively).

**Table 2:** Subject characteristics of the patients who quitted smoking.

	Asymptomatic smokers	Smokers with asthma	Chronic bronchitis/COPD
**Subjects (n)**			
**3 mo**	6	4	7
**6 mo**	4	4	1
**Female/Male**			
**3 mo**	3/3	3/1	4/3
**6 mo**	3/1	3/1	1/0
**Age yrs**			
**3 mo**	47 ± 6	46 ± 2	59 ± 11
**6 mo**	50 ± 5	46 ± 2	57
**Pack-yrs**			
**3 mo**	15 ± 10	21 ± 18	36 ± 14
**6 mo**	19 ± 11	21 ± 18	21
**Post-bronchodilator**			
FVC L			
**3 mo**	4.2 ± 0.65	3.7 ± 0.68	2.9 ± 0.48
**6 mo**	3.9 ± 0.67	3.7 ± 0.68	3.5
FVC % pred			
**3 mo**	95 ± 6	99 ± 27	85 ± 9
**6 mo**	96 ± 6	99 ± 27	83
FEV1 L			
**3 mo**	3.5 ± 0.68	3.0 ± 0.53	2.1 ± 0.43
**6 mo**	3.2 ± 0.71	3.0 ± 0.53	2.35
FEV1% pred			
**3 mo**	96 ± 5	98 ± 26	75 ± 8
**6 mo**	97 ± 6	98 ± 26	70
FEV1/FVC			
**3 mo**	83 ± 4	82 ± 2	71 ± 7
**6 mo**	82 ± 5	82 ± 2	82 ± 2

**Figure 1:**
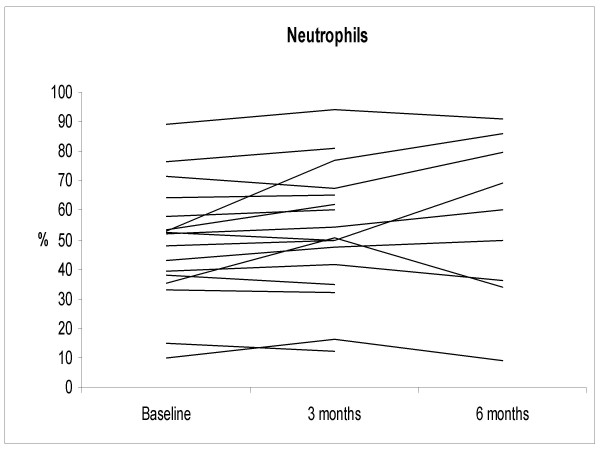
**The percentage of induced sputum (IS) neutrophils in the smoking cessation group (n = 17, see Table 2) at the onset of smoking cessation (baseline) and at 3 and 6 months after stopping smoking**. IS neutrophils were significantly higher in the smoking cessation group at the baseline compared to the non-smokers (p = 0.021). Neutrophils increased significantly from baseline at 3 months after cessation (p = 0.035) and declined to the levels of non-smokers at 6 months after the cessation.

**Figure 2:**
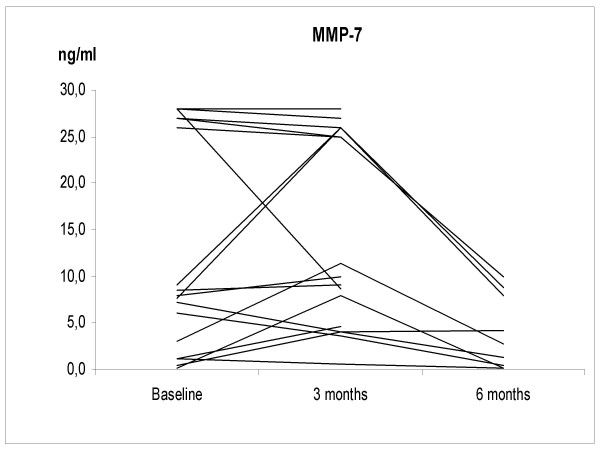
**The levels of MMP-7 in the smoking cessation group at the baseline and at 3 and 6 months after stopping smoking**. The levels of MMP-7 were significantly higher in the smoking cessation group at the baseline compared to the non-smokers (p = 0.014). MMP-7 declined to the levels of non-smokers at 6 months after the cessation.

**Figure 3:**
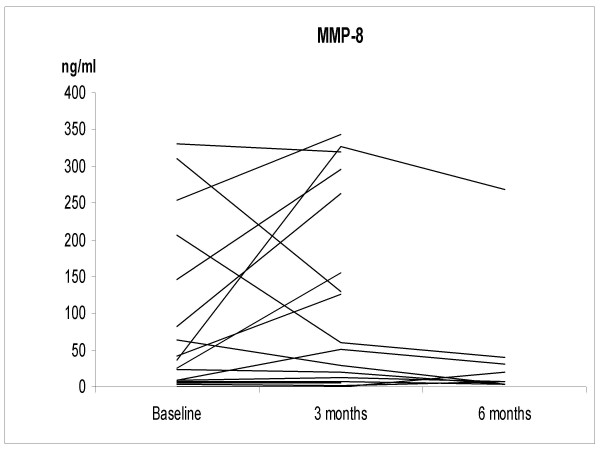
**The levels of MMP-8 in the smoking cessation group at the baseline and at 3 and 6 months after stopping smoking**. The levels of MMP-8 were significantly higher in the smoking cessation group compared to the non-smokers (p = 0.001). MMP-8 declined to the levels of non-smokers at 6 months after the cessation.

**Figure 4:**
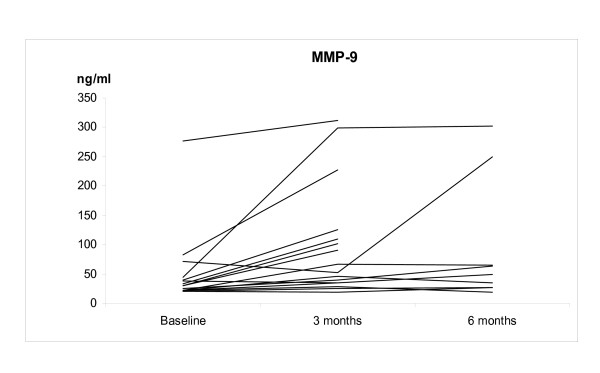
**The levels of MMP-9 in the smoking cessation group at the baseline and at 3 and 6 months after stopping smoking**. The levels of MMP-9 were significantly higher in the smoking cessation group compared to the non-smokers (p = 0.02). MMP-9 levels remained elevated in the smoking cessation group when compared to the levels of non-smokers at 6 months after the cessation (p = 0.017).

**Figure 5:**
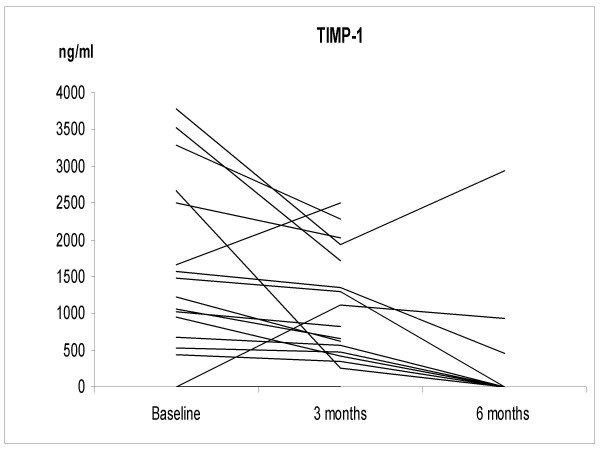
**The levels of TIMP-1 in the smoking cessation group (n = 17) at the baseline and at 3 and 6 months after stopping smoking**. The levels of TIMP-1 were significantly higher in the smoking cessation group compared to the non-smokers (p = 0.006). TIMP-1 declined to the levels of non-smokers at 6 months after the cessation.

The IS neutrophil proportions increased significantly at 3 months after smoking cessation; 67.5 ± 13.7%, 2.9 ± 2.6 × 10^6 cells/ml (p = 0.035), but at 6 months the difference from non-smokers was no longer statistically significant (38.2 ± 14.4%, 0.74 ± 0.83 × 10^6 cells/ml) (Figure 1). The individual levels of MMP-7, -8 and TIMP-1 at 3 months after cessation did not change significantly from the corresponding baseline levels of these same subjects. The corresponding individual levels after smoking cessation at 6 months decreased significantly compared to the baseline (p = 0.032, p = 0.001, p = 0.04, respectively) (Figures 2, 3, 4 and 5). They also decreased nearly to the levels of non-smokers in the cross sectional evaluation, though there was high variability in the TIMP-1 levels. In contrast, the individual levels of MMP-9 increased significantly from the baseline at 3 months after cessation (p = 0.009) and did not differ significantly at 6 months after the cessation when compared to the baseline levels in these same individuals (p = 0.069), and the differences were even more significant when compared to the non-smokers in a cross sectional evaluation (p = 0.017).

## Discussion

As far as we are aware, this is the first prospective study to examine how smoking cessation is reflected in the levels of sputum MMPs. Most previous smoking cessation studies have been cross-sectional and the real effects of smoking cessation on airway remodelling processes/tissue destruction have remained unclear. Based on this study, it seems that sputum MMP levels generally decline slowly after smoking cessation. However, the elevated levels of MMP-9 in smokers are sustained for many months after giving up smoking for reasons that remain unclear.

Smoking cessation reduces COPD morbidity, hospital admissions [25] and COPD progression [1]. It has also been shown that symptoms decrease significantly after a month of quitting of smoking [8]. The decline of lung function is attenuated slowly, only after 2-3 years from smoking cessation [26,27]. Smoking evokes very similar airway pathology in asthma and COPD, with the typical features being the recruitment of increased numbers of neutrophils to the airways and elevated oxidative stress [4,8]. In this study, smoking resulted in neutrophil predominance and elevation of several MMPs in the IS of smokers with or without mild airway disease as compared to the situation in non-smokers. The fact that MMP-9 increased from the baseline during the 3-6 months after smoking cessation suggests that extracellular matrix breakdown continue in the airways for several months after an individual stops smoking. Since neutrophils are an important source of MMP-9, it could be argued that the elevated level of this MMP is simply a marker of neutrophilic inflammation. However, the finding of the persistently elevated MMP-9 levels after 6 months at a time when the neutrophil counts had returned to those encountered in non-smokers suggests that the rise in MMP-9 is not dependent on the numbers of neutrophils per se but instead reflects increased MMP-9 release. Our recent studies have shown that the elevated levels of MMP-9 are associated with increased MMP-9 activity [16,28]. However, Lowrey et al. [29] have reported that MMP-9 protein but not MMP activity is higher in sputum of smokers with COPD when compared to smokers without COPD. The significance of MMP-9 in the airways needs further investigations, since it has also been suggested that MMP-9 may have protective role at least against ozone induced airway inflammation [30]. It needs to be emphasized also that our results are preliminary and final conclusions about the significance of sputum MMP-9 after smoking cessation remain unclear.

All of the other markers studied had returned to the levels of non-smokers by 6 months after cessation, which suggests that significant repair of tissue damage elicited by smoking has been initiated. A previous study noted that inflammatory markers such as macrophages and IL-8 declined significantly in asymptomatic smokers one year after smoking cessation [31]. This is in agreement with our results showing that MMP-7, -8 and TIMP-1 though its' variability can return to normal levels within 6 months after the patients successfully quits smoking. Baseline proportion of neutrophils and the levels of MMPs and TIMP-1 were significantly higher in smokers compared to those of non-smokers. These findings are in full agreement with the studies reporting that MMP-8, -9 and TIMP-1 are elevated in COPD and that the increased levels are related to smoking [10,16,32] and also that the disease itself, not only smoking, results in MMP elevation [33-35]. MMP-7 is induced by hypoxia [36] but, as far as we are aware, this is the first study showing that MMP-7 elevation is provoked by smoking. MMP-7 is expressed by macrophages and expression is upregulated also in pulmonary epithelial cells in the presence of chronic infection, which might support the hypothesis that MMP-7 contributes to pulmonary immunity [37] and that smoking cessation decreases the chronic inflammation in the airways.

One weakness of this study was the high number of dropouts, despite our best efforts, only 15% of our subjects were still non-smoking six months later. This considerable numbers of the dropouts was to be anticipated taking into account the well-known difficulties associated with smoking cessation. In the study of Kaper et al. [38], for example, subjects receiving reimbursed smoking cessation treatment had an abstinence rate of 5.5% half a year after the interventions. In the present study, the cases from different subgroups were combined, which was also justified for several reasons i.e. the subjects were smokers who had no airway disease or the disease was mild. Smoking asthmatics also develop airway neutrophilia in a similar manner as smokers and subjects with COPD, and the same phenomenon was found in this study. Moreover, the differentiation of symptomatic smokers from mild COPD, and smoking asthmatics from COPD is a demanding task since there is a clear overlap in the cell profiles and diseases [4,8]. In our study, also the MMP levels were overlapping in these subgroups of smokers as expected. The high number of dropouts diminishes the power of this study, why these results, though important, require further investigations with larger numbers of patients with various phenotypes of asthma and COPD.

## Conclusions

In summary, our study clearly suggests that the high levels of MMP-9 in the airways persist for at least 6 months after smoking cessation, and this does not appear to be related to the numbers of inflammatory cells in the airways. It is still unclear whether MMP-9 is one of the instigators to the ongoing disease progression in some individuals after smoking cessation or whether it has a protective role in airway inflammation.

## Competing interests

The authors declare that they have no competing interests.

## Authors' contributions

NL participated in the recruitment of non-smokers and smokers, and writing the manuscript. HS and WM helped with the statistics and writing. PR participated in the selection and recruitment of the smokers. VLK and RD participated in the design and coordination of the study, and writing of the manuscript. All authors have read and approved the final manuscript.

## Pre-publication history

The pre-publication history for this paper can be accessed here:

http://www.biomedcentral.com/1471-2466/10/13/prepub
